# What is the qualitative evidence concerning the risks, diagnosis, management and consequences of gastrointestinal infections in the community in the United Kingdom? A systematic review and meta-ethnography

**DOI:** 10.1371/journal.pone.0227630

**Published:** 2020-01-17

**Authors:** Suzanne Rotheram, Jessie Cooper, Sara Ronzi, Benjamin Barr, Margaret Whitehead

**Affiliations:** 1 Health Protection Research Unit in Gastrointestinal Infection (NIHR), Farr Institute @ The Health eResearch Centre, University of Liverpool, Liverpool, United Kingdom; 2 Department of Public Health & Policy, The University of Liverpool, Liverpool, United Kingdom; 3 Division of Health Services Research and Management, School of Health Sciences, University of London, London, United Kingdom; National Center for Global Health and Medicine, JAPAN

## Abstract

**Background:**

Gastrointestinal (GI) infections cause a significant public health burden worldwide and in the UK with evidence pointing to socio-economic inequalities, particularly among children. Qualitative studies can help us understand why inequalities occur and contribute to developing more effective interventions. This study had two aims: 1. Conduct a systematic review to determine the extent and nature of UK qualitative evidence on gastrointestinal infections; 2. Use meta-ethnography to examine the influences of the differing social contexts in which people live.

**Methods:**

MEDLINE, Scopus, Web of science, CINAHL and JSTOR were searched for UK qualitative studies engaging with the risk, diagnosis, management or consequences of gastrointestinal infections from 1980 to July 2019. Five reviewers were involved in applying inclusion and exclusion criteria, extracting and synthesising data (PROSPERO CRD 42017055157).

**Results:**

Searches identified 4080 studies, 18 met the inclusion criteria. The majority (n = 16) contained data relating to the risk of gastrointestinal infection and these made up the main synthesis. The tenets of meta-ethnography were used to glean new understandings of the role of social and environmental contexts in shaping the risk of gastrointestinal infection, specifically with respect to foodborne GI illness. Three main explanations concerning risk emerged from the data: explanations of risk in the community were underpinned by understandings of ‘bugs’, dirt and where food comes from; risks were negotiated in households alongside diverse processes of decision making around food; and resources available to households shaped food practices.

**Conclusion:**

This systematic review highlights the scarcity of UK qualitative evidence examining gastrointestinal infections. Apart from risk, questions around diagnosis, management and consequences of illness were largely untouched. No studies investigated patterning by socio-economic status. Nevertheless, the meta-ethnography yielded wider contextual theories and explanations as to *why* people might not follow food hygiene guidance, giving pointers to the types of qualitative enquiry needed to develop more effective interventions.

## Introduction

Gastrointestinal (GI) infections are a group of illnesses which are largely characterised by symptoms of vomiting and/or diarrhoea often accompanied by abdominal pain and fever [1]. They can be caused by a variety of agents, for example, bacteria, viruses, parasites and toxins, which can be transmitted in multiple ways [2]. These routes of transmission include through food or water, person-to-person spread, through the environment or through contact with animals [2].

GI infections are an important public health issue worldwide. While in many cases the symptoms of vomiting and/or diarrhoea caused by these illnesses are mild and self-limiting, they can result in more severe consequences, particularly for children and frail elderly people [3]. A World Health Organisation study examining the global burden of 22 foodborne gastrointestinal diseases in 2010 estimated there to be 582 million cases each year resulting in 25.2 million Disability Adjusted Life Years (DALYs) [4]. In the United Kingdom (UK) it has been estimated that around one in four people in the UK suffer with a GI infection each year [2]. This group of illnesses therefore cause a significant public health burden in the UK in terms of individual morbidity and financial costs to families, the economy, and the National Health Service (NHS) [2,5]. The two most common bacterial and viral pathogens causing illness are estimated to cost patients in the UK £114.6 million (through lost income, medication and childcare) and the NHS £16 - £22 million each year [5].

This review looked at UK qualitative studies examining GI infections. This included *foodborne gastrointestinal illness* (GI illness which occurs when pathogens or toxins are consumed in food or water) and studies which examined aspects of *non-foodborne GI infections* (GI infections transmitted by routes other than food, for example, person-to-person spread) [2].

The majority of UK research literature in the field of GI infections in the community, including analyses of socio-economic inequalities in infection, focuses on describing the risk and pattern of disease using epidemiological data (for examples see [2,5–8]). A recent systematic review points to the social patterning of GI infections with higher rates found in children living in more disadvantaged socio-economic conditions [9]. Further epidemiological studies found that the consequences of a GI infection in terms of illness severity and time off work was greater for poorer populations of all ages [10]. While these epidemiological studies describe patterns of inequalities, they do not explain why these patterns are observed or the processes leading to different outcomes for different groups.

Many practitioners and organisations collaborate to prevent and control GI infections in the UK. These include (but are not limited to): Public Health England (PHE), the Food Standards Agency (FSA), the NHS, Environmental Health Officers, water companies, abattoirs, farmers and businesses selling or providing food. Foodborne GI illness prevention is addressed at a structural level by UK legislation and regulation to prevent pathogens and toxins entering the food chain [11]. Once food enters the home, however, there is a tendency for health campaigns to take a *narrow*, *individualistic* approach to prevention by focusing on the responsibility of individuals to modify their behaviours around food, without considering the economic, social or cultural context of people’s lives. Campaigns to prevent foodborne illness, for example, have included: the *‘simple 4Cs principles of good hygiene’*, highlighting the responsibility of individuals to clean, cook, avoid cross contamination and chill food correctly; the promotion of the correct use of date labels on food; and a campaign urging people to stop washing chicken [12,13]. The same focus on the responsibility of individuals can also be found in advice produced by PHE and reiterated in NHS guidance to prevent GI infections spread person-person in the community. This advises people to: stay away from work and school for 48 hours after symptoms have passed; to avoid visiting General Practitioners (GPs); and to use good hand hygiene [14]. Despite these concerted efforts, rates of GI infections remain persistently high [2,15].

We argue that the current attempts to modify individual behaviours as a way of tackling the level of GI infections is too narrow in its approach. Instead, we suggest following the lead of research on GI infections in Low and Middle Income (LMI) countries, which examine the wider social and structural contexts shaping these infections. The use of ethnographic approaches in LMI settings, for example, have demonstrated that issues such as diverse understandings of illness within communities, inadequate sanitation and access and availability of clean water interact with, and shape GI infections [16–18]. Such an approach provides insight into the complexities of GI infections which, in combination with epidemiological analyses, may assist in developing interventions focusing on broader structural, rather than individual, behavioural change [19].

The systematic review reported here was conceived to determine the extent and nature of the existing qualitative evidence in the UK on GI infections in the community. In a second step, we aimed to take the synthesis further, by employing a meta-ethnographic approach to capture the bigger picture. Meta-ethnography is an interpretative approach to the synthesis of all types of qualitative research which allows for the development of concepts across studies using different understandings of cultural expression and theoretical perspectives [20–23]. The authors used the tenets of meta-ethnography and took a social constructionist approach which recognised that the meanings, experiences and practices of GI infections will be constructed by different people in different ways depending on their interaction with other people within their social context [24]. In so doing, the authors were able to capture the theories and explanations about the possible influences of the differing social and economic contexts in which participants lived. The ultimate goal was to inform the development of more effective interventions around the risk and management of GI infections in the UK.

## Methods

The systematic review followed the Preferred Reporting Items for Systematic Reviews and Meta-analysis (PRISMA) guidelines [25], was registered with the International prospective register of systematic reviews (PROSPERO) database (PROSPERO CRD 42017055157) and addressed the following review question:

*What is the extent and nature of the qualitative evidence for the risks*, *diagnosis*, *management or consequences of gastrointestinal infections in the community in the United Kingdom*?

### Search strategy, selection criteria and screening

The search strategy was developed using terms for GI infections used in a related systematic review [9]. These were combined with terms to describe qualitative research and the UK and piloted in a scoping search developed in MEDLINE (S1 File). We searched five electronic databases (MEDLINE, Scopus, Web of Science core collection, CINAHL and JSTOR) and sources of grey literature (Open Grey, ProQuest dissertations and theses). We also hand-searched the FSA report repository foodbase [26], research projects catalogued on the FSA website [27], reference lists of identified studies and contacted experts in the field to identify any additional sources. Searches were run in July 2019 and were restricted to studies from 1980 to July 2019 in the UK to ensure that the findings were relevant to the current public health and policy context with regards to GI infections in the UK. Studies not in the English language were excluded as the authors did not have the necessary expertise to design and implement multi-lingual searches [28]. The inclusion and exclusion criteria are shown in Table 1.

**Table 1:** Inclusion and exclusion criteria.

**Inclusion criteria**
Studies that collect primary qualitative data or, in the case of a mixed methods study, include a qualitative aspect that has been analysed using a qualitative method of analysis.
Persons of any age or gender.
Studies that report on any aspect of the risks, diagnosis, management or consequences of GI infections.
Studies conducted in the community with people who are living independently and not in institutions.
Studies conducted in the UK.
Studies published from 1980 to July 2019.
Studies published in English language.
**Exclusion criteria**
Studies that do not collect primary qualitative data. Quantitative studies, surveys and opinion pieces are therefore excluded.
Studies that do not report on an aspect of the risks, diagnosis, management or consequences of GI infections.
Studies not conducted in the community. Studies based in institutions, e.g. hospitals, are excluded. Studies focused on food hygiene regulations, implementation and management practices within businesses are also excluded.
Studies reporting on GI infections as sequelae to treatment (e.g. immunosuppressive drugs) for another condition are excluded.
Studies conducted outside the UK.
Studies published before 1980.
Studies not published in English language.

Retrieved papers were exported into EndNote reference manager [29] where duplicates were removed. One reviewer (SRotheram) screened titles and abstracts for eligibility in EPPI-Reviewer 4 [30] using a pre-piloted tool developed using the criteria in Table 1. Two reviewers (SRonzi and AP) independently screened 10% of titles and abstracts. Full text papers were screened in the same way. Any differences were resolved by discussion or referral to a third reviewer (JC or MW).

### Data extraction and quality assessment of included studies

One reviewer (SRotheram) extracted data from included studies using a pre-piloted form containing the reference details, the type, aim and location of the study, qualitative data collection methods and participant characteristics (Table 2) [31]. Data were only extracted for direct quotes and ethnographic observations cited in study reports [32]. These were entered into NVivo 10 software for analysis. For the one study including participants from multiple countries, only UK quotes were extracted [33].

**Table 2:** Study grading using checklist taken from the National Institute for Health and Care Excellence (NICE) quality appraisal checklist for qualitative studies [34].

Grading	Quality assessment criteria
**++**	All or most of the checklist criteria have been fulfilled, where they have not been fulfilled the conclusions are very unlikely to alter.
**+**	Some of the checklist criteria have been fulfilled, where they have not been fulfilled, or not adequately described, the conclusions are unlikely to alter.
**-**	Few or no checklist criteria have been fulfilled and the conclusions are likely to alter

Quality assessment was completed by SRotheram. A second reviewer (MW) completed quality assessments for one in four of the papers to ensure consistency in the use of the criteria. Quality assessment was completed using the National Institute for Health and Care Excellence (NICE) quality appraisal checklist for qualitative studies [34,35]. Each study was graded as outlined in Table 2.

### Data synthesis

The broad focus of this review question yielded a wide range of published studies. A narrative synthesis was therefore adopted, which is an appropriate methodology for synthesising qualitative research which employed diverse methods [31]. The synthesis involved an application of three steps performed in an iterative way with two reviewers (SRotheram, JC) going backwards and forwards between steps.

Step i) a preliminary synthesis of the findings and quality assessment of the included studies. Textual descriptions and tables were used to organise studies, determine how the studies were related [20,36] and relate the studies as a whole to particular aspects of the review. The studies were assessed for quality using the NICE checklist which grades the studies according to a cumulative scoring system [34]. This tool uses a cumulative scoring system to assess: how appropriate a qualitative research approach is to the research questions; the relevance and rigor of the methodological approach; and the adequacy of the findings [34,35].
Step ii) an exploration of the relationships between, and a re-interpretation of the primary data from the included studies. Spider diagrams were used to visually explore relationships in the data and were used alongside thematic analysis of extracted data conducted by two reviewers (SRotheram and JC) using NVivo 10 [31]. The extracted data were read and re-read to identify codes inductively across the data. All members of the research team discussed these codes and grouped them into broad areas of similarity (translations) which still retained the spirit of the individual studies [21]. This analysis then went one step further, drawing on the tenets of meta-ethnography to facilitate translation between studies and move beyond the original interpretation of the primary data [21]. This approach was used to capture the possible influences of differing social contexts in which people live [20,21]. The initial codes (translations) were reduced and integrated to develop a line-of argument synthesis to capture the possible influences of differing social contexts. In so doing, we were able to identify new relationships and themes not identified in the original studies [20,21,23,37].
Step iii) an assessment of the robustness of the synthesis produced. The NICE quality appraisal was used as the basis for the assessment of the robustness of the synthesis. An assessment of the overall strength of the evidence available is addressed in the discussion [31].

## Results

### Study selection and characteristics

**Fig 1:**
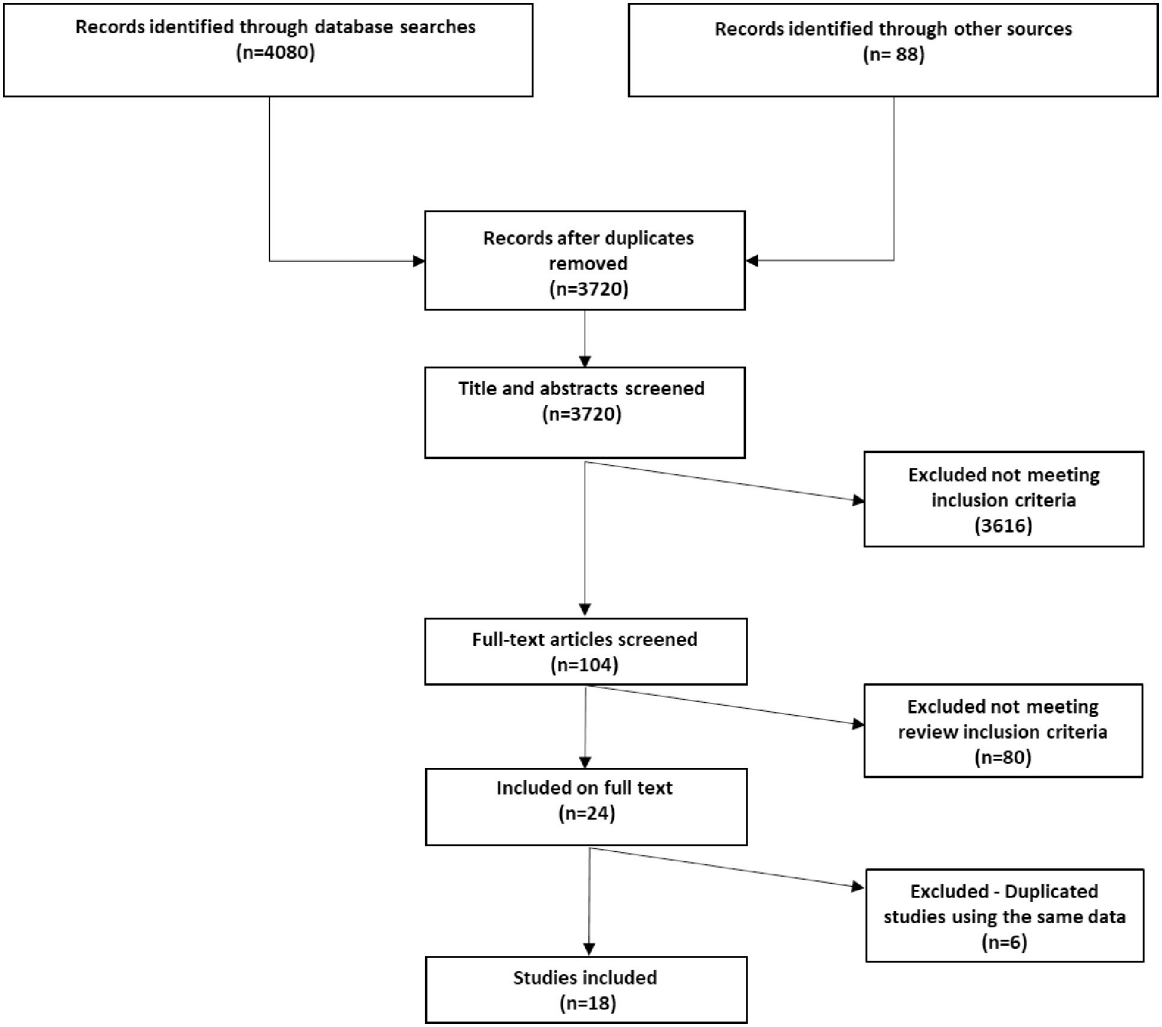
PRISMA diagram for searches from 1980 to July 2019. Study selection process.

Fig 1 shows the PRISMA flow diagram of the study selection process. From the 4080 papers identified in the original searches, 24 met the inclusion criteria. Of the 24 studies 11 were unique studies and were therefore included. Four were primary studies with six additional publications from these primary studies which replicated data and findings from the original study. The six publications which replicated data from the primary studies were excluded and only the four primary studies were included in the analysis. The remaining three studies were companion articles which originated from the same study but presented different data and findings. All three of these companion studies were included in the analysis making a total of eighteen studies included in the review (see Fig 1 and Table 3).

**Table 3:** Summary details of studies including: Reference details; type of study and location; aim of study; data collection methods; participant characteristics; whether the study engages with the diagnosis/risk/management/consequences of illness and quality appraisal.

Author(s) and date	Type of Study Location	Aim of study	Data collection methods Participant characteristics	Risk/Diagnosis/Management/ Consequences	Quality appraisal [34]
Albon (2010)	Qualitative London	*‘To develop a greater understanding of food and drink practices in order to encourage early years’ practitioners to examine*, *re-appraise and improve their practice*.*’*	Observations, ethnographic and semi-structured interviews. Semi-structured interviews—28 staff in 4 early childhood settings. Observations—staff and children aged 0–5 years in 4 settings.	Risk	++
Curtis et al. (2003)	Mixed-methods Wirral, UK	*‘To pinpoint particular risk practices and to understand what motivates domestic hygiene behaviour*.*’*	Semi- structured interviews, projective interviews & focus group. Participants: carers from 10 households with one child under 3 months and one under 3 years. Semi-structured interviews—5 carers. Projective interviews—5 carers. 1 x focus group—5 carers.	Risk	+
Enticott (2003)	Qualitative Village in NW Devon, population 324	*‘To understand why*, *at a time of constant concern over food safety*, *some consumers continue to consume ‘risky’ foods*.*’*	Ethnographic methods—formal & ethnographic interviews and participant observations. Semi-structured interviews– 25 village members.	Risk	+
Evans (2011)	Qualitative South Manchester	*‘To explore the ways in which households plan for and shop for food; how they prepare*, *consume and eat it; how they store it and how they dispose of the food they do not eat*.*’*	Ethnographic methods—repeat in-depth interviews, ‘hanging out’ in homes and streets, diary records, ‘going along’ with shopping trips and as participants prepare food, cupboard rummages, fridge inventories, kitchen and home tours. 19 households: mix of income, age, housing structure, tenure and composition.	Risk	+
Eves et al. (2010)	Mixed-methods Schools in SE England	*‘To determine knowledge of food hygiene amongst young children (5–7 years) and facilitators and barriers to the application of knowledge*.*’*	In-depth interviews. 30 children, 5–7 years.	Risk	+
Green et al. (2003)	Qualitative Urban & rural SE England.	*‘To focus on how participants account for ‘choosing safe food’*.*’*	Focus groups. 11 focus groups in the UK: adolescents, young single consumers, family food purchasers and consumers 55+.	Risk	+
Lugg (2014)	Mixed-methods England & Wales	*‘To explore the experience*, *management and beliefs surrounding a paediatric gastroenteritis episode from a clinical & lay perspective*, *the beliefs behind the variation and the possible impact this had on clinicians*, *parents and patients*.*’*	Semi-structured telephone interviews. 28 female parents with a child under 5 who has had acute gastroenteritis in the last 3 months. Clinicians– 18 General Practitioners responsible for managing paediatric gastroenteritis.	Risks, Management & Consequences	++
Meah & Watson (2011) ^1^	Qualitative South Yorkshire & Derbyshire	*‘To explore the ways in which differing—and often competing -discourses and sources of knowledge regarding cooking and food safety practice knowledge have been negotiated into everyday routines*.*’*	Focus groups, formal interviews, ethnographic methods: provisioning ‘go alongs’, videos of kitchen tours and meal preparation. 7 x focus groups. 20 interviews from 7 families.	Risk	+
Meah (2014) ^1^	Qualitative UK	*‘To explore how perceptions of risk and responsibility are rationalised by participants on a range of different levels*, *sometimes resulting in practice that might be regarded by food safety experts as ‘risky’ or ‘dangerous’*.*’*	Focus groups, formal interviews, ethnographic methods: provisioning ‘go alongs’, videos of kitchen tours and meal preparation. 7 x focus groups. Household study: representatives of 2–4 generations from 8 families across 17 households.	Risk	+
Milne (2011)	Qualitative Sheffield and Norfolk.	*‘To explore the attitudes and routines related to food in general and participants’ use of food labelling allowing participants to concentrate on their own concerns*, *or lack of*, *about food*, *including but not limited to safety*.*’*	Focus groups. 6 x focus groups with members of the public: ages 60–90 (n = 34).	Risk	+
Redmond & Griffith (2013)	Mixed-methods	*‘To obtain qualitative data from consumers and relative caregivers concerning beliefs*, *attitudes and practices relating to infant feeding with powdered infant formula inside and outside the home*.*’*	Focus groups. Focus groups: Parents x7. Health visitors x3. Nursery employees x3. Hospital nurses x3.	Risk	+
Shaw (2001)	Qualitative. South of England.	*‘To explore expert and lay understandings of food risks*.*’*	Semi-structured interviews. 17 with ‘experts’ involved in food related work. 32 with ‘lay’ people; parents with young children, older people, young people, organic food eaters, vegetarians and members of the farming community.	Risk	++
Van Kleef et al. (2006)	Qualitative UK	*‘To understand how food risk management practices are perceived amongst various relevant stakeholder groups with an interest in food safety*.*’*	Focus groups. 1 x food consumer focus group. 3 x ‘expert’ focus groups.	Risk	++
Watson et al. (2013) ^1^	Qualitative South Yorkshire & Derbyshire	*‘To explore the tensions that arise in public discourses around food safety*, *thrift*, *saving and reuse around provisioning as ‘stuff’ crosses the line between food and waste*.*’*	Focus groups and ethnographic methods. Food focused life history interviews, observations, ‘go alongs’ on shopping trips, videos and photos of kitchen tours and meal preparation. Interviews—23 participants from 17 households. Ethnographic work with 15 households.	Risk	+
Wills et al. (2013)	Qualitative UK	*‘To examine practices in the kitchen to assess where and how such practices have the potential to influence food safety in the home*.*’*	Ethnographic approach: kitchen tour and mapping exercise, photography and photo-elicitation, observation and video-observation, informal interviews, use of diaries and scrap books. 20 UK households, 10 with people over 60 years, 2 with pregnant women.	Risk	++
Wythe (2015)	Mixed-methods Hertfordshire and Milton Keynes	*‘To relate personal history*, *health and demographic contexts to the accumulation of food hygiene assets throughout the life course and the pre-disposing conditions that might impact upon asset mobilisation*.*’*	Semi-structured interviews. Older people in sheltered accommodation (n = 15), wardens (n = 3?).	Risk	+
Lecky et al. (2014)	Qualitative Gloucester	*‘To explore the barriers to stool sample collection and specimen return to ascertain which factors may help to improve the process*.*’*	‘Flexible’ interview. 26 patients 31–70 years old.	Diagnosis	++
McNulty et al. (2012)	Qualitative Gloucestershire	*‘To determine what criteria GPs use to decide when to send stool samples*, *what (including National guidance) informs these decisions*, *and their opinion of the National guidance available*.*’*	Telephone interviews and discussion group. Telephone interviews with 20 GPs: varying stool submission rates–high, average and low. Discussion event with 22 GPs from 19 surgeries.	Diagnosis & Management	++

^1^ Companion articles—presenting different data and findings from the same research study.

Eighteen studies conducted in the UK, published between 2001 and 2014 were included in the review (Table 3). While not all these studies stated the investigation of the risk, diagnosis, management or consequences of gastrointestinal infection in the community as an objective, all contained data relevant to the review question in their findings. Most studies included participants from a variety of age groups. Two studies included children [38,39] and two focused on older people [40,41]. Seven studies also included professionals such as experts with a food safety role [33,42], community GPs [43,44], wardens working in community sheltered accommodation for the elderly [40] and professionals involved in early childhood care in the community [39,45]. The majority of studies (n = 11) did not detail the ethnicity of their participants. Of the remaining studies, four included participants with a diverse range of ethnicities [38,39,46,47] and three stated that the majority of their participants were of White British descent [40,48,49].

Studies incorporated a variety of approaches, with many studies integrating multiple qualitative methods including: in-depth interviews (n = 12); focus groups (n = 8); and ethnographic approaches incorporating observations and informal interviews (n = 7) (Table 3). Most studies (n = 16) included data related to the risks of illness, with one study also incorporating data related to its management and consequences [43]. Two studies included data relating to the diagnosis of illness [44,47] and one study contained data relating to both diagnosis and management [44] (Table 4). Fourteen studies included data related to foodborne GI illness alone, while four studies included data which could relate to both foodborne GI illness and non-foodborne GI infections [43,44,47,50] (Table 4).

**Table 4:** Details of whether the study was related to foodborne GI illness or non-foodborne GI infections & risk/diagnosis/management/consequences of illness.

First author & date	Risk/Diagnosis/Management/Consequences	Foodborne GI illness/Non-foodborne GI infection
Albon (2010)	Risk	Foodborne
Curtis et al. (2003)	Risk	All
Enticott (2003)	Risk	Foodborne
Evans (2011)	Risk	Foodborne
Eves et al. (2010)	Risk	Foodborne
Green et al. (2003)	Risk	Foodborne
Lugg (2014)	Risks, Management & Consequences	All
Meah & Watson (2011)^1^	Risk	Foodborne
Meah (2014)^1^	Risk	Foodborne
Milne (2011)	Risk	Foodborne
Redmond & Griffith (2013)	Risk	Foodborne (infant formula)
Shaw (2001)	Risk	Foodborne
Van Kleef et al. (2006)	Risk	Foodborne
Watson et al (2013)^1^	Risk	Foodborne
Wills et al. (2013)	Risk	Foodborne
Wythe (2015)	Risk	Foodborne
Lecky et al. (2014)	Diagnosis	All
McNulty et al. (2012)	Diagnosis & Management	All

^1^ Companion articles—presenting different data and findings from the same research study.

### Quality appraisal

Tables 3 and 5 outline the quality appraisal of included studies. The implications that this quality assessment has on the trustworthiness of the review is discussed in the conclusions. The aspects of quality that were the least well demonstrated included the description of the role of the researcher, a lack of clarity in the rigor of the data analysis and the reporting of ethics (Table 5). No studies were assessed as being of poor quality. The review therefore did not include studies with poor methodological quality [31].

**Table 5:** Quality appraisal of included studies.

First author, year published	Is a qualitative approach appropriate?	Is the study clear in what it sets out to do?	How defensible/rigorous is the research design methodology?	How well was the data collection carried out?	Is the role of the researcher adequately described?	Is the context clearly described?	Were the methods reliable?	Is the data analysis sufficiently rigorous?	Is the data ‘rich’?	Is the analysis reliable?	Are the findings convincing?	Are the findings relevant to the aims of the study?	Conclusions.	How clear and coherent is the reporting of ethics?	Overall assessment
**Albon (2010)**	Appropriate	Clear	Defensible	Appropriately	Clearly described	Clear	Reliable	Rigorous	Rich	Not sure	Convincing	Relevant	Adequate	Appropriate	++
**Curtis et al. (2003)**	Appropriate	Clear	Defensible	Appropriately	Clearly described	Clear	Reliable	Not sure	Poor	Not sure	Convincing	Relevant	Adequate	Not reported	+
**Enticott (2003)**	Appropriate	Clear	Defensible	Appropriately	Not described	Clear	Reliable	Not reported	Rich	Not reported	Convincing	Relevant	Adequate	Not reported	+
**Evans (2011)**	Appropriate	Clear	Defensible	Inadequately reported	Not described	Clear	Reliable	Not reported	Rich	Not reported	Convincing	Relevant	Adequate	Not reported	+
**Eves et al. (2010)**	Appropriate	Clear	Defensible	Appropriately	Not described	Clear	Reliable	Not reported	Poor	Not reported	Convincing	Relevant	Adequate	Not reported	+
**Green et al. (2003)**	Appropriate	Clear	Defensible	Appropriately	Not described	Clear	Reliable	Not reported	Rich	Not reported	Convincing	Relevant	Adequate	Not reported	+
**Lecky et al. (2014)**	Appropriate	Clear	Defensible	Appropriately	Clearly described	Clear	Reliable	Rigorous	Rich	Reliable	Convincing	Relevant	Adequate	Appropriate	++
**Lugg (2014)**	Appropriate	Clear	Defensible	Appropriately	Not described	Clear	Reliable	Not sure	Rich	Reliable	Convincing	Relevant	Adequate	Appropriate	++
**McNulty et al. (2012)**	Appropriate	Clear	Defensible	Appropriately	Clearly described	Clear	Reliable	Rigorous	Rich	Reliable	Convincing	Relevant	Adequate	Appropriate	++
**Meah & Watson (2011)**	Appropriate	Clear	Defensible	Inadequately reported	Not described	Clear	Reliable	Not reported	Rich	Not reported	Convincing	Relevant	Adequate	Appropriate	+
**Meah (2014)**	Appropriate	Clear	Defensible	Inadequately reported	Not described	Clear	Reliable	Not reported	Rich	Not reported	Convincing	Relevant	Adequate	Not reported	+
**Milne (2011)**	Appropriate	Clear	Defensible	Appropriately	Not described	Clear	Reliable	Rigorous	Rich	Not reported	Convincing	Relevant	Adequate	Not reported	+
**Redmond & Griffith (2013)**	Appropriate	Clear	Defensible	Appropriately	Not described	Clear	Reliable	Not reported	Rich	Not reported	Convincing	Relevant	Adequate	Appropriate	+
**Shaw (2001)**	Appropriate	Clear	Defensible	Appropriately	Clearly described	Clear	Reliable	Rigorous	Rich	Reliable	Convincing	Relevant	Adequate	Not reported	++
**Van Kleef et al. (2006)**	Appropriate	Clear	Defensible	Appropriately	Unclear	Clear	Reliable	Rigorous	Rich	Reliable	Convincing	Relevant	Adequate	Not reported	++
**Watson et al. (2013)**	Appropriate	Clear	Defensible	Appropriately	Clearly described	Clear	Reliable	Not reported	Rich	Not reported	Convincing	Relevant	Adequate	Not reported	+
**Wills et al. (2013)**	Appropriate	Clear	Defensible	Appropriately	Clearly described	Clear	Reliable	Rigorous	Rich	Reliable	Convincing	Relevant	Adequate	Appropriate	++
**Wythe (2015)**	Appropriate	Clear	Defensible	Appropriately	Not described	Clear	Reliable	Not sure	Rich	Not sure	Convincing	Relevant	Adequate	Appropriate	+

### Overview of study content

As most studies (n = 16) contained data relating to *risks* of GI infections these formed the basis of the further synthesis and re-interpretation in this review (Tables 3 & 4) [23,36]. Of these 16 studies, 14 contained data only relating to foodborne GI illness, so it is the qualitative data relating to the risk of foodborne GI illness which is examined in this review.

These 16 included studies took a range of perspectives and theoretical approaches to their research aims and objectives (Table 4): three studies did not aim to examine the risk of foodborne GI illness explicitly but engaged with food risks in their results [39,43,51]; five studies explored participants’ knowledge, understanding and attitudes towards food safety [33,38,42,45,52]; and one examined individual home hygiene practices [50]. The remaining studies looked to understand why members of the public do not follow ‘expert’ guidelines and advice on ‘safe’ food practices [40,41,46,48,49,53,54]. Many included studies took an individual approach to the risk of GI infections and no studies explored observed inequalities in GI infections or experiences across different socio-economic groups.

From these 16 studies, engaging with the risks of foodborne GI illness, nine codes (translations) were created. All of these were informed by multiple papers (see Table 6). After discussion amongst the research team, 3 further codes were developed which, taken together, give overarching insights into what qualitative research can tell us about the risk of GI infections spread through food in the community in the UK (Table 6).

**Table 6:** Formation of codes (translations).

Codes (translations)	No. of contributing items of evidence	No. of contributing papers	Overarching insights
Bugs are good for you	18	10	Risk underpinned by understandings of bugs, dirt and where food comes from
Food groups influencing safety concerns	21	7	
Beliefs around inherent safety of food	41	7	
Origin & preparation of food shaping safety concerns	30	6	
Using experience & acquired knowledge	176	15	Risk negotiated alongside diverse processes of decision making around food
Other people shaping practices	87	14	
Considerations of food waste	24	7	
Individual concerns about finances or wasting money on food	37	7	The availability of resources shape practices around food
Organisational & service constraints	33	5	

### What does existing UK qualitative research tell us about the risk of foodborne GI illness?

Three overarching insights were identified.

1. The risk of foodborne GI illness was underpinned by participants’ understandings of ‘bugs’, dirt and where food comes from.
2. These risks were negotiated alongside diverse processes of decision making around food.
3. The availability of resources shapes practices around food.

The synthesis of the primary data in these studies provided insights into how practices around food were underpinned by a variety of understandings about ‘bugs’ and dirt and what makes food ‘safe’. The term ‘practice’ used in this way simply refers to what people do, their ‘arrays of activity’ [55]. The term ‘bug’ used in this way refers to a lay term for ‘pathogen’. This synthesis and re-interpretation also drew out how practices around food were shaped by wider contextual factors, such as the social settings in which people live and the resources available to individuals and organisations.

#### Understandings underpinning the risks of foodborne illness

Thirteen studies contained data describing how the risk of foodborne GI illness was underpinned by understandings of ‘bugs’, dirt and where food comes from [33,38,52–54,39,40,42,43,45,46,48,49]. In stark contrast to public health food safety messages promoting the elimination of dirt and germs [56,57], data demonstrated that participants interpreted that exposure to certain ‘bugs’, in particular circumstances, was protective [33,39,40,42,45,46,52–54], building up their natural immunity. For example, the ‘germs’ in raw (unpasteurised) milk were thought to increase resistance to other diseases:

*Raw milk—it’s OK—you get some germs but you get a better resistance to all these diseases—you build up some antibodies*.

(Consumer of unpasteurised milk) [53]

Exposure to dirt and ‘bugs’ seemed to be considered particularly important for building up a child’s immune system [33,39,43,45]. Parents explained that they preferred their children to *‘have a few germs’ and that it was possible to be ‘too clean’* (parent focus group) [45]. These potential ‘risks’ of over-cleanliness extended to using antibacterial cleaning products which were perceived to reduce immunity to everyday ‘bugs’ [33,42,45,52,54]. Older participants explained that they thought that illnesses were now more common as a direct result of living in a more hygienic environment [42,46]:

*You do need a certain amount of dirt*, *bacteria and things*, *if you kill it all off everybody’s immune system is up the swanny… So there has to be a balance… and I think nature*, *left to herself*, *gets it right*. *When you begin to tip it then you run into all kinds of trouble*.

(Member of Over 50s lunch club) [42]

Despite this appreciation of ‘bugs’ and dirt in the environment and food, not all were understood to be ‘good’. The data did not illuminate how participants distinguished between ‘good’ and ‘bad’ dirt and ‘bugs’, but a majority of studies contained data recognising dirt and ‘bugs’ as ‘bad’ and therefore an important consideration when deciding if food was ‘safe’ to eat [33,38,52,54,39,40,42,45,46,48,49,51]. Participants who believed that contamination with dirt or pathogens in the production process meant that food was not inherently ‘safe’ when you bought it [33,38,42,45,48,51] would take precautions to prevent foodborne illness by washing, cooking or heating food with care [33,38,42,45,48]. A health visitor who said that she understood that powdered milk was not sterile explained that this was why she advised parents to follow the preparation recommendations:

‘*that’s why the water had to be hot*, *because when the powder hits it*, *it actually sterilises the powder for that feed*.’

(nursery nurse) [45]

Judgements as to whether food was ‘safe’ were also shaped by considerations of hygiene and dirt according to who had prepared it, where it came from, or the type of food. Food prepared by children, in certain homes, shops and markets were perceived to be ‘dirty’, less ‘safe’ and therefore to be avoided [39,42,45,48,52,54]:

*On Christmas party day in setting one*, *families bought in food from home to share with the group as a whole*. *One family had made a plate of sandwiches*, *which were not touched by the staff*, *indeed the practitioners went out of their way to warn each other which foods to avoid*. *Mary stated ‘God I wouldn’t eat anything from there*. *It was filthy on home visit*.’

(Early childhood practitioner) [39]

In contrast, food bought from a trusted butchers, produced locally, or with a label of ‘organic’ or ‘free range’ was thought to be lower ‘risk’ and therefore ‘safer’ to eat [42,48,52]. In some cases, participants explained that their views had led them to change shopping habits to buy food from people or places believed to be ‘safer’ and therefore less likely to make them ill [42,48,52].

Particular food groups seen as unsafe often correlated with food safety awareness campaigns, although the source of these beliefs were not always clear to participants. Chicken, pork, uncooked meats, fish, eggs (especially in the studies conducted around the UK Salmonella epidemic (1997–1998)) [58], ready-made meals, rice and take-away meals were considered ‘high risk’. Again, these assessments could prompt changes in shopping habits as well as storing, preparation and cooking of these food groups [40,42,45,46,48,51,52]:

*No*, *it was probably a few something like a few days gone so I ‘umm-ed’ and ah-hed about it but it’s chicken so you’ve got to be careful… and I wasn’t going to risk it as you’re going to know about it if you eat bad chicken*.

(Faye, 20s) [51]

This theme suggests that people may assess whether food is ‘good’ or ‘bad’ and ‘safe’ or ‘unsafe’ to eat using a variety of diverse and contrasting beliefs. It demonstrates how ‘bugs’ in food and dirt might not always be considered ‘bad’ and how these assessments can then be used to inform what people do around food.

#### The process of decision-making around food

All 16 studies illustrated how decisions and practices around food were shaped by participants’ own experiences, acquired knowledge or interactions with other people. Acquired formal knowledge about food safety from school, food hygiene courses or public health messages could shape what people did around food [38,40,42,45,46,48,49,51,52]. Participants also explained, however, that they deliberately went against public health recommendations because they had previously chosen not to follow this advice and had no adverse consequences [42,45,46,48,52,53]. Examples included: drinking unpasteurised milk [53], eating food past its use by date [48], consuming runny egg yolks while pregnant [42,48], making up bottles of powdered baby milk without using current guidelines [45] and re-heating rice or meat multiple times [46]:

*Stuart (41)*, *for example*, *used to work in the food industry as a dairy manager*, *which perhaps gives him some insider ‘know-how’*. *He reports having reheated boeuf bourguignon five times in the past*, *but that his wife wouldn’t ever allow that now … [whispers] it’s all a load of rubbish I think … some people say you shouldn’t heat meat more than twice*, *re-heat it*, *but I’ve done it three or four times I’m still here*, *I’m fine*. *So’s Sally [wife]*, *so’s the kids*.

(Stuart, 41) [46]

Contrasting with this finding, an experience of illness could also be used to decide what to eat and how to cook food [42,43,48,52,53]:

*I’ve had food poisoning twice in my life*, *and it was so bad that it scared me… I was taken ill on pâté… so because I was taken ill I never touch pâté*, *because it stays in your mind*.

(Member of lunch club, 68–85) [42]

In addition to this acquired knowledge and experiences, studies reported that participants drew on their senses such as smell, taste and the visible look of food to help them decide if food was safe to eat and to rationalise eating things past their sell-by date [33,38,52,54,40,42,45,46,48–51]:

.. *[People] don’t understand what [these dates] mean* … *I say to people*, *‘Do you think that this use-by date … today it’s not a problem*? *Is it a problem tomorrow*? *It will kill you*, *is that what you think*? *What do you think this use-by date*, *it is the day that’s set well ahead of some possible danger that it might have’* … *In principle* … *I generally ignore these dates*, *completely ignore them*, *and I look at them and*, *depending on how it looks and how it tastes*, *how it smells and it’s*, *it won’t kill you if you have a taste*, *and if the taste isn’t very good you can throw it away*.

(Ted, 66) [46]

Other people could influence the way things were done either through advice or simply by cohabiting and sharing the same space [38,39,50,52–54,40–43,45,46,48,49]. Activities around food were shaped by a myriad of people and negotiated between multiple members of the household [45,48,49,52] with one of the most prominent influences on how people handled food being intergenerational interactions within families. Older relatives shaped what their children or grandchildren did with food, passing practices from one generation to the next [38,40,45,46,54]. Some practices remained unchanged throughout peoples’ lives. An 83-year old participant described how she still treated milk near its use by date in the same way as her mother treated milk that the milkman had delivered that was warm after having sat on the doorstep for the day:

***R***: *Mum would say some nights ‘Mmm’ because the milk would come in the morning anyway*, *first thing*. *‘Mmm that milk is not very cold’ she used to scald it*, *so it didn’t quite bring it to boiling point but just underneath*, *and that would keep until next day then so that was all right* …

***I***: …*oh that’s interesting I have not heard that one before*.

***R***: *I still do it myself sometimes*.

***I***: *Do you*?

***R***: *Yeah I think ‘oh that milk is out of date tomorrow perhaps I will just do that before* … *(sentence ends)*.

(Ruth, 83 years) [40]

The younger generation could also influence the older generation’s food practices either directly or indirectly. Children might, for example, re-arrange their parent’s fridge or throw away out of date food [41,49,52]. Alternatively, the perceived vulnerability of children or elderly adults could also change cooking practices indirectly by making people more cautious when preparing food for them [42,43,45,48–50,52]:

*When I’ve got my baby great-grand-daughter here… I’m very careful how I cook the egg*, *I cook it longer*, *or poach it*, *rather than lightly boil it* … *I would be more nervous… anything to do with food poisoning*, *that’s why I’m fussy with the baby* … *you can’t be too careful… I won’t risk anything like that*.

(Member of lunch club, 68–85)[42]

These findings suggest that participants’ own experiences alongside the influence of other people may play an important role in decision making around food practices. Moreover, as we show next, the resources that are accessible to people and organisations may also be a contributing factor in how people treat food.

#### The availability of resources shapes what people do around food

Nine studies included data relating to how the availability of resources within households or organisations might shape practices around food [33,40–42,45,48,49,52,53]. Financial constraints within households prevented people from buying food they perceived to be ‘safer’ (but more expensive) or influenced their decisions about whether to throw away food past its best before date [40,41,45,48,49,52]. Concerns around wasting food were particularly evident amongst elderly participants who had experienced, first or second hand, growing up in a time of austerity when food was in scarce supply [41,48,49]:

… *the idea of wasting*… *I mean that’s like a thread right through from you know*, *being a kid after the war*, *you just didn’t waste anything*. *It’s always like a big worry about food*, *in terms of food hygiene*, *it’s the idea of not letting your food go off ‘cause then you’d waste it*.

(Laura, 64) [49]

Shortage of time was reported by service providers to limit their opportunities to educate about, and practise, safe food preparation [33,40,45]. Professionals working within community services with new parents explained that, with regards to staffing levels, *‘resources were very stretched’* (Health visitor) [45] and could restrict communication to parents on how to safely prepare a bottle of infant milk formula:

‘*We haven’t got time; and also it’s not an appropriate venue because most of us are doing sort of postnatal weigh ins and things*, *so it’s not a venue that you can actually discuss sterilising with*, *there are just too many coming through*.’

(Health visitors) [45]

Time pressures could also restrict the time available for carers to support elderly people in food safety practices:

…*carers will come in*, *they will grab the first thing*, *obviously they have got a time restraint*, *‘oh we will grab that one we will use that milk first’*, *whereas really they should have used the other one first*, *but they haven’t looked at that*, *so a lot of it is observation basically*.

(Warden in sheltered accommodation) [40]

Physical restrictions on space or access to working fridges could influence food safety by forcing people to store food in fridges that did not keep food at the recommended temperature, or areas of the house not meant for food [45,48,49]:

*For example*, *in some of the study households*, *lack of available storage space meant that participants stored items such as drinks*, *tinned and dried goods and vegetables in under-stairs cupboards*, *the garage*, *utility rooms*, *bedrooms*, *a downstairs shower cubicle or even a relative’s home*.

(Fieldwork notes)[48]

Elderly participants who had grown up with no access to fridges and freezers described how, when they were young, they would shop locally and eat food on the day of purchase [40–42]. The movement of shops out of town centres alongside health-related deterioration in mobility restricted access to food, which in turn affected how elderly participants stored food, with the freezer acting as a vital resource [40,41]:

*I use the freezer a lot*, *always for meat*, *I’ve always got some frozen vegetables in case I need them but I’ve got fresh as well*, *and I use it for bread*. *If you can’t do your shopping*, *I mean Ted can do some of it but I don’t like him doing too much because of his heart and you’ve always got something in then if you can’t get to the shops or the homecare workers don’t come*. *I’m worse now than I used to be at hoarding food because it’s become a real issue*, *if you can’t get food*, *it’s very difficult*.

(Focus group member of the public) [41]

The data suggest that the availability of resources, restrictions on organisations and changes to local and wider environments might influence the actions of individuals and organisations around food, offering an important consideration for reducing levels of foodborne GI illness.

## Discussion

To our knowledge this is the first systematic review to identify the extent and nature of the qualitative evidence in the UK on risks, diagnosis, management and consequences of gastrointestinal infections. It is also the first to re-examine the findings of individual studies on this subject using meta-ethnography to draw out contextual insights.

As a whole, the review serves to highlight the sparsity of the qualitative evidence base in the UK on the subject of GI infections: only 18 studies in the UK between 1980 and July 2019 met our inclusion criteria. The majority of this research (16 out of 18 studies) focused on the *risk* of GI infections, leaving questions around the diagnosis, management and consequences of illness largely untouched. Furthermore, as most of the studies (14 out of 18 studies) focused solely on the risk of foodborne GI illness, UK qualitative research currently gives us few insights into GI infections spread by other routes such as person-to-person. No study investigated why or how socio-economic inequalities in GI infections might come about.

Strikingly, many identified studies took an individualistic approach, restricting interpretation of findings to matters of individual choice and possible lack of adherence to public health messages around buying and preparing food. Our narrative synthesis and re-interpretation of the data using meta-ethnography, however, reveal deeper understandings of why and how people make decisions which influence their risk of foodborne illness. Importantly, the re-interpretation of findings identified evidence that people’s beliefs, actions and decisions around food may also be shaped by the social, cultural and economic circumstances in which they live, suggesting that what people do with food may be better understood as social-structural processes [59]. The findings of this review provide a different perspective to the current UK public health approach to the risk of foodborne GI illness, which has a tendency to focus on practices around food as a consequence of individual choice and responsibility, in isolation from the wider context in which people live [60].

### Strengths and limitations

The findings of this study are limited by the small number of qualitative studies and the restricted scope of some of these studies. Despite extensive searching, we cannot be certain we identified all relevant published papers as the indexing of qualitative research within databases is recognised as making searching for these studies particularly challenging [61]. This small body of research, however, scored well on the quality assessment and no poor quality studies were included. This increases the trustworthiness of the findings as its conclusions are based on credible findings in the primary studies [31].

The findings of the review are also limited to the understandings which can be gained through analysing the quotes and observations selected by the authors of the original studies rather than the full dataset [21,22]. The selection of these quotes within primary studies may have been influenced by the particular methodological perspective or theoretical approach of these authors which may, therefore in turn impact on the outcome of this synthesis [22]. In keeping with the social constructionist approach of this review, however, rather than attempting to providing a complete understanding, this study can be seen as enriching the current insights into the experiences and practices of managing the risk of foodborne illness within their social context [22].

The aim of this review was to contribute to understandings of gastrointestinal infections within the context of UK policy. While doing an analysis specific to one context is in keeping with a meta-ethnographic approach this limits the study’s transferability to other countries [36]. There may, however, be some transferability to other contexts which take a similar policy approach to the UK.

These limitations aside, the synthesis and re-interpretation of data in this review gives useful insight into the ‘problem’ of the risk of GI infections, within an understanding of the wider contextual elements shaping food practices. A next step would be to follow the example of previous research and use the insights gained from this review alongside statistical methods to quantify its findings [62,63]. This could be done with a survey of a representative sample from the population, the results of which could inform future interventions to reduce the incidence of foodborne illness.

#### Insights for development of future food hygiene interventions

Even within the limited scope of the studies that we identified (risks associated with individual food hygiene behaviour), this modest body of evidence offers some useful insights into *why* people do not always follow food hygiene guidance. It also gives pointers to the types of qualitative enquiry that are needed to develop more effective interventions concerning risk in the future. The studies reveal, for example, a variety of diverse and contrasting beliefs about whether food is ‘good’ or ‘bad’ and ‘safe’ or ‘unsafe’ to eat, which in turn can influence whether people follow or apparently go against standard food hygiene advice.

The evidence also showed how decisions and practices around food were sometimes shaped by interactions with other people–not least intergenerational interactions within families. This raises further possibilities for knowledge exchange, over and above the common practice of focusing on the person doing most of the food preparation in a household. Public information campaigns need to be more aware that what people do around food might be influenced by interactions with other people and consider directing interventions to the community, organisational and environmental levels rather than continuing to focus messages at individual behaviour change [64,65].

A few studies provided glimpses of the ways in which socioeconomic circumstances might shape how people follow public health advice related to use- by- dates and food storage. These included: financial constraints restricting the purchase of food considered to be ‘safer’ and influencing decisions about whether to throw away food past its best before date; physical restrictions on space and poor food-storage facilities limiting safe storage of food; and greater distances to shops meaning that people store food for longer. Budget cuts to NHS and local authority services added additional restrictions on the availability of advice and help on food safety. These examples underline the need for greater sensitivity to the socio-economic context in which people live in any actions to try to change food practices. The limited number and scope of these studies, however, restricts the insights that can be gained for wider prevention strategies. Such strategies require both broader and deeper qualitative enquiry, as outlined below.

#### Implications for future qualitative research

The gaps in the evidence base identified in this systematic review emphasise the further qualitative research needed to take forward the prevention agenda in this field. Future research is needed to explore the contextual elements identified in the review further and contribute to a more robust evidence base for these findings. In doing so, an approach that takes on board ‘lay knowledge’ is important. ‘Lay knowledge’ around food may develop over time, using experiences, life events and other sources alongside information from ‘experts’ [66]. It has been argued in other areas of public health that ‘lay knowledge’ can contribute to health practitioners’ understanding of health, illness and risk [67–69]. The use of lay knowledge around food could be an additional but essential source of expertise for public health professionals looking to find ways to reduce foodborne illness. One way to access this knowledge within the community could be through the use of community participatory research [70,71].

Nearly all the studies we reviewed were concerned with *risk* of GI infections, specifically foodborne GI illness, yet the examination of the *management* and *consequences* of GI infections may be particularly important to understanding socio-economic differences in infection rates, because of the more severe consequences of GI infections for more disadvantaged groups [10]. Further research is needed on GI infections which are not transmitted through food, which make up the majority of GI infections in the UK each year [7,15]. Social science methodologies, such as ethnography, could be utilised effectively to examine contextual as well as individual factors that might influence the risk and transmission of GI infections not caused by food. The social context of infections spread person-person within communities may be a particularly relevant area to explore using these methods.

Finally, it is important to examine how inequalities in GI infections are generated and maintained. While research identified in this review includes a focus on vulnerable groups such as children and frail elderly people, none of the qualitative studies examined why the observed differences by socioeconomic status outlined in the introduction to this paper come about [9,10]. This consideration of the wider context is crucial to both understand why these inequalities exist and what can be done to reduce them. To do this, the research needs to go beyond the individual, health education level, taking a multi-layered approach to include community, organisational and environmental interventions to reduce such inequalities [65,72].

## Supporting information

S1 File Search terms used in Medline. (DOCX)

S1 Table PRISMA checklist. (DOC)
