# Correction: What is the qualitative evidence concerning the risks, diagnosis, management and consequences of gastrointestinal infections in the community in the United Kingdom? A systematic review and meta-ethnography

**DOI:** 10.1371/journal.pone.0230468

**Published:** 2020-03-12

**Authors:** The PLOS ONE staff

There is an error in affiliation 3 for Jessie Cooper. The correct affiliation 3 is: Division of Health Services Research and Management, School of Health Sciences, City, University of London, London, England, United Kingdom. The publisher apologizes for the error.
