# Supplementary material for: What is the qualitative evidence concerning the risks, diagnosis, management and consequences of gastrointestinal infections in the community in the United Kingdom? A systematic review and meta-ethnography
Source: PLoS One. 2020 Jan 17;15(1):e0227630. doi: 10.1371/journal.pone.0227630 (PMC6968854; doi:10.1371/journal.pone.0227630)
Supplement: S1 File — (DOCX) [file pone.0227630.s001.docx]

# S1 File

## **Example of search strategy/syntax**

**MEDLINE** (ALL) (multi-purpose/unqualified search) ran via OVID 10-07-2019. Limited to English Language and humans and 1980-2019 (week 28)

1. exp Foodborne Diseases/
2. Food poisoning*.mp.
3. Food hazard*.mp.
4. exp Food Handling/
5. Kitchen practice*.mp.
6. Food polic*.mp.
7. exp Food Safety/
8. exp Food Contamination/
9. Food risk*.mp
10. Hygeine practice*.mp.
11. exp Hand Hygiene/
12. faecal-oral transmission.mp.
13. exp Fluid Therapy/
14. oral rehydration.mp.
15. stool collection*.mp.
16. stool specimen*.mp.
17. enteric infection*.mp
18. acute gastroenteritis.mp.
19. infectious intestinal disease*.mp.
20. gastrointestinal infection*.mp.
21. exp Gastroenteritis/
22. exp Gastrointestinal Diseases/
23. exp Diarrhea/
24. diarrh*.mp.
25. stomach flu.mp.
26. gastric flu.mp.
27. stomach bug*.mp.
28. stomach virus.mp.
29. winter vomiting disease*.mp.
30. exp Dysentery, Bacillary/
31. gastrointestinal pathogen*.mp.
32. gastrointestinal bacteria*.mp.
33. exp Norovirus/
34. exp Rotavirus/
35. exp Rotavirus Vaccines/
36. exp Campylobacter/
37. exp Escherichia coli/
38. exp Enterobacteriaceae Infections/
39. exp Escherichia coli Infections/
40. exp Yersinia enterocolitica/
41. exp Salmonella Infections/
42. exp Cryptosporidiidae/
43. exp Salmonella/
44. exp Shigella/
45. exp Giardia/
46. exp Listeria/
47. Small round structured virus*.mp.
48. exp Sapovirus/
49. exp Caliciviridae/
50. VTEC.mp.
51. exp Shiga-Toxigenic Escherichia coli/
52. Scombro*.mp.
53. exp Clostridium perfringens/
54. Exp Bacillus cereus/
55. Hepatitis A.mp.
56. Hepatitis E.mp.

#57 OR 1 to 56.

#58. exp Qualitative Research/

#59. phenomenology.mp.

#60. exp Interview/

#61. exp Hermeneutics/

#62. exp Anthropology cultural/

#63. exp Anthropology/

#64. exp Anthropology Medical/

#65. ethnograph*.mp.

#66. exp grounded theory/

#67. exp Focus Groups

#68. exp Observation/

#69. visual research.mp.

#70. exp personal narratives/

#71. exp sociology/

#72. exp Sociology Medical/

#73. exp Social Sciences/

#74. OR 58 to 73

#75. exp Great Britain/

#76. #57 AND #74 AND #75

#77. Limit 76 to English language and humans and years=’1980-2019’

#78. Limit 76 to English language and humans and years=‘1980-2016’
